# The tropical coral *Pocillopora acuta* displays an unusual chromatin structure and shows histone H3 clipping plasticity upon bleaching

**DOI:** 10.12688/wellcomeopenres.17058.1

**Published:** 2021-07-29

**Authors:** David Roquis, Céline Cosseau, Kelly Brener Raffalli, Pascal Romans, Patrick Masanet, Guillaume Mitta, Christoph Grunau, Jeremie Vidal-Dupiol

**Affiliations:** 1 Agroscope, Nyon, 1260, Switzerland; 2 IHPE, Univ. Montpellier, CNRS, Ifremer, Univ. Perpignan Via Domitia, Montpellier, France; 3 Observatoire Océanologique de Banyuls, Paris, France; 4 Aquarium de Canet-en-Roussillon, Canet-en-Roussillon, France

**Keywords:** Pocillopora acuta, Pocillopora damicornis, invertebrate epigenetics, chromatin structure, Histone H3 clipping

## Abstract

**Background:** *Pocillopora acuta* is a hermatypic coral with strong ecological importance. Anthropogenic disturbances and global warming are major threats that can induce coral bleaching, the disruption of the mutualistic symbiosis between the coral host and its endosymbiotic algae. Previous works have shown that somaclonal colonies display different levels of survival depending on the environmental conditions they previously faced. Epigenetic mechanisms are good candidates to explain this phenomenon. However, almost no work had been published on the *P. acuta *epigenome, especially on histone modifications. In this study, we aim at providing the first insight into chromatin structure of this species.

**Methods:** We aligned the amino acid sequence of *P. acuta* core histones with histone sequences from various phyla. We developed a centri-filtration on sucrose gradient to separate chromatin from the host and the symbiont. The presence of histone H3 protein and specific histone modifications were then detected by western blot performed on histone extraction done from bleached and healthy corals. Finally, micrococcal nuclease (MNase) digestions were undertaken to study nucleosomal organization.

**Results:** The centri-filtration enabled coral chromatin isolation with less than 2% of contamination by endosymbiont material. Histone sequences alignments with other species show that *P. acuta* displays on average ~90% of sequence similarities with mice and ~96% with other corals. H3 detection by western blot showed that H3 is clipped in healthy corals while it appeared to be intact in bleached corals. MNase treatment failed to provide the usual mononucleosomal digestion, a feature shared with some cnidarian, but not all; suggesting an unusual chromatin structure.

**Conclusions:** These results provide a first insight into the chromatin, nucleosome and histone structure of *P. acuta*. The unusual patterns highlighted in this study and partly shared with other cnidarian will need to be further studied to better understand its role in corals.

## Introduction

Epigenetic modifications are good candidates to explain rapid, inheritable and reversible phenotypes without changes in the deoxyribonucleic acid (DNA) sequence ( Danchin *et al.*, 2011). They range from chemical modifications of DNA ( *e.g.* cytosine methylation), covalent changes of proteins participating to chromatin structure ( *e.g.* histone modifications), as well as nuclear localization of chromosomes, and non-coding RNAs involved in post-transcriptional silencing of genes and repeated regions. These modifications have an impact on the chromatin structure, leading to the modulation of transcriptional activity ( Wu & Morris, 2001). It is now clear that environmental factors can influence the epigenome to induce the expression of new phenotypes ( Duncan *et al.*, 2014; Feil & Fraga, 2012).

In natural populations with genetic diversity, disentangling the respective roles of genetics and epigenetics in environmentally triggered phenotypes is hardly feasible, if not impossible, except with biological models with asexual reproduction. This rare feature is one of the characteristics of colonial organisms, such as corals, and they have been used as a model to address such questions. Most of these studies are focused on DNA methylation ( Dixon *et al.*, 2018; Liew *et al.*, 2018) and non-coding ribonucleic acid (ncRNA) ( Huang *et al.*, 2017; Huang *et al.*, 2019; Liew *et al.*, 2014), probably because the methods available to study these epigenetic marks are more straightforward. To date, only one article on Scleractinia corals has been performed at the histone modification level and has studied the broad nucleic content of the H2A.X and its phosphorylated form gamma-H2A.X ( Rodriguez-Casariego *et al.*, 2018). Many technical challenges make the epigenome analysis difficult: extraction of cells and tissues from the stony exoskeleton, working at a marine salt concentration to maintain cell, protein and chromatin integrity, and most importantly, separation of coral and Symbiodiniaceae biological material. Symbiodiniaceae are endocellular, and to focus on the coral epigenetic modifications, we can focus on non-symbiotic life stages, use naturally or artificially bleached colonies, or use laboratory techniques enabling physical separation of symbionts and host’s nuclei and genetic material.

In this article, we describe a novel method to isolate coral nuclei (and by extension, coral chromatin) of *Pocillopora acuta*. We chose this coral for its worldwide distribution and ecological importance ( Hoeksema *et al.*, 2015), its fast growth, allowing to quickly generate new colonies through propagations ( Hughes *et al.*, 2015), and its documented response to thermal stresses ( Vidal-Dupiol *et al.*, 2009; Vidal-Dupiol *et al.*, 2014). *P. acuta* has a genome size of approximately 325 Mb ( Vidal-Dupiol *et al.*, 2019), while Symbiodiniaceae genome size is estimated at ~1,200–1,500 Mb ( Lin *et al.*, 2015; Shoguchi *et al.*, 2013), which means that a single Symbiodiniaceae brings as much DNA as five coral cells, hence the necessity to reduce contamination from endosymbiotic material at the lowest possible level. We then used the isolated coral nuclei for the analysis of histone modifications and provide a first insight about the chromatin structure of this species. We also show that rupture of symbiosis (bleaching) is associated with changes in histone H3 structure (H3 clipping).

## Methods

### Ethics approval

The *Direction Départementale de la Cohésion Sociale et de la Protection des Populations* (DDSCPP) provided the permit N°C66-136-01 to IHPE for experiments on animals. Housing, breeding and animal care were done following the national ethical requirements ( https://eur-lex.europa.eu/legal-content/EN/TXT/HTML/?uri=CELEX:32010L0063).

### Biological material

The *Pocillopora acuta* isolate used in the present study was harvested in Lombock (Indonesia, CITES number: 06832/VI/SATS/LN/2001) and maintained at the Banyuls Aquarium (France) under optimal conditions (~26°C, pH 8.2, NO _3_<0.1 mg/L, PO _4_<0.01 mg/L, Ca ^2+^ ~450 mg/L, Mg ^2+^ ~1350 mg/L, KH ~7, PAR ~150 µmol photon/m²/s, 12h/12h light dark photoperiod, water motion of 30X tank volume per hour). Previously assigned to *Pocillopora damicornis*, this isolate was reassigned to *P. acuta* ( Vidal-Dupiol *et al.*, 2019). Bleached coral colonies were obtained through a menthol treatment. Briefly, the colonies (5 colonies of ~5 cm in diameter) were placed in a four-liter tank filled with seawater. Water motion was created using a submerged water pump (set at ~100 L/h, EHEIM Compact ON 300), temperature was maintained at 27°C and light adjusted to 75 µmol/m²/s (PAR). Corals were bleached by menthol treatment. The protocol for the menthol treatment was adapted from Wang *et al.* (2012). The first day, the corals were subjected to a menthol concentration of 0.58 mmol/L for 6h. After this exposure, they were transferred to the coral nursery for a 18h recovery period. During the second day, the same protocol was applied (menthol treatment and recovery step). The third day, the corals were exposed again to the same treatment but only until the polyps were closed. Once the polyps were closed the corals were placed in the coral nursery for recovery and Symbiodiniaceae loss. This last step typically takes four to five days. In this experiment, the coral colonies were left to bleach for five days. Once the polyps open up again, colonies were sampled. Healthy and bleached coral fragments were immediately frozen and stored in liquid nitrogen.

In this work, we also employed other *Cnidaria* for comparison in the chromatin extraction and digestion experiments. We chose three other hermatypic corals, *Stylophora pistillata* (CITES number IAZ3924), *Montipora digitata* (CITES number IEZ0069) and *Euphyllia divisa* (CITES number IUZ1609). We also used *Aiptasia sp*. and *Anemonia manjano*, two sea anemones ( *Cnidaria* phylum), sharing an endosymbiodic relationship with Symbiodiniaceae, but without an aragonite skeleton. All these species were acquired from the Canet en Roussillon aquarium (France). All of the species we used in this study are part of the Hexacorallia subclass. The anemones are from the *Actinaria* order and the corals from the *Scleractina* order. Symbiodiniaceae cultures ( *Cladocopium* sp. [type1] and *Fugacium* sp.) were obtained from the Roscoff Culture Collection (strain CCMP 2246, cat# RCC4017).

### *In silico* identification of canonical histones

We were interested in using isolated coral chromatin to perform chromatin immunoprecipitation (ChIP) on histone modifications. Before starting experiments, we wanted to confirm that histones were present in *P. acuta* genome, and that their amino acid sequences would be similar enough to other metazoan to be able to target them with commercial antibodies. Using Mega X ( Kumar *et al.*, 2018) with the ClustalW algorithm under default parameters, we aligned the *P. acuta* predicted histone protein sequences ( Vidal-Dupiol *et al.*, 2019) to reference protein sequences from *Mus musculus*, *Schistosoma mansoni*, a platyhelminth for which our laboratory has extensive experience in ChIP, and four other Cnidaria: *Pocillopora damicornis*, *Acropora digitifera*, *Nematostella vectensis* and *Hydra vulgaris*. Both *P. damicornis* and *A. digitifera* are from the same Scleractinia order as *P. acuta*. Accession numbers for each species are in Table 1.

**Table 1.** Histone protein sequences accession numbers. List of accession numbers of canonical histones amino acids sequences used for multiple alignment. Sequences for *Pocillopora acuta* come from the recently assembled genome (see Vidal-Dupiol *et al.*, 2019 for the complete data).

	H2A	H2B	H3	H4
***P. acuta***	TCONS_0002941	TCONS 00004949	TCONS_00016826	TCONS_00024865
***P. damicornis***	XP_027055899.1	XP_027035879.1	XP_027053709.1	XP_027055898.1
***A. digitifera***	XP_015747526.1	XP_015757821.1	XP_015766372	XP_015748868.1
***N. vectensis***	XP_001626711.1	XP_001617661.1	XP_001623766.1	XP_001630070.1.1
***H. vulgaris***	XP_002158325.1	XP_002156085.2	XP_002154470.1	XP_004205459.1
***S. mansoni***	XP_018651265.1	XP_018644054.1	XP_018646598.1	XP_018647790.1
***M. musculus***	AAA37763.1	NP_783594.1	NP_038576.1	NP_291074.1

### Western blots

Histones were either acid-purified with the Abcam histone extraction kit (cat #ab113476) from bleached and healthy coral fragments of approximately 5 mm of diameter (~ 0.5 g), or, for rapid extraction, coral fragments were put into 1.5 mL tubes filled with 500 μL of a solution containing 62.5 mM TRIS/Cl pH 6.8, 3% SDS, 10% sucrose, 0.2 M dithiotreitol (DTT) and 1.25 mM sodium butyrate. Tubes were sonicated using a Vibra Cell 75185 at 70% amplitude, 3 times 15 seconds, on ice. A cooldown on ice of 30 seconds was done between each sonication. *Dinoflagellata* phylum, which includes Symbiodiniaceae, possess core histones, but they have been documented to be larger and with different lateral chains than most metazoan ( Marinov & Lynch, 2015). In previous experiments, we systematically failed to obtain a signal from in western blots with commercial anti-histone antibodies on Symbiodiniaceae. Knowing this, we decided to use Symbiodiniaceae histone extraction as a negative control (to be certain the signal we observe in healthy and bleached *P. acuta* truly comes from the coral and not its symbiotes). We chose hamster ( *Mesocricetus auratus*) as a positive control, as most antibodies are commercially developed and tested on mammals. Hamster brain and cultured aposymbiotic Symbiodiniaceae protein extracts were processed the same way as *P. acuta*. The laboratory has permission A 66040 from both French *Ministère de l’Agriculture et de la Pêche* and *French Ministère de l’Education Nationale de la Recherche et de la Technologie* for experiments on animals and certificate for animal experimentation (authorization 007083, decree 87-848 and 2012201-0008) for the experimenters. Housing, breeding and animal care follow the national ethical requirements.

The extract was cleared by centrifugation for 30 minutes at 1500 g, and the supernatant was collected. Total proteins (5 mg per sample) were mixed with Laemmli buffer (final concentration 1X) and denatured at 99°C for 5 mins. ECL™ Full-Range Rainbow (Amersham cat# RPN800E) was used as a molecular weight marker. Protein separation was done on 10% SDS-PAGE gel electrophoresis before being blotted on a nitrocellulose membrane (Trans-Blot turbo, Bio-Rad). The membrane was blocked with 5% non-fat dry milk in tris-buffered saline containing 0.05% tween 20 (TBST) one hour at room temperature. One of the following primary antibodies, diluted in 5% non-fat milk in TBST was used for each western blot: anti- histone H3 (Abcam cat# ab1791 lot# 784471, polyclonal, dilution 1/500), anti-H3K4me3 (Millipore cat# 04-745 lot# 2326991, monoclonal, dilution 1/1000), anti-H3K9ac (Millipore cat# 07-352, lot# 2203126, polyclonal, dilution 1/500), anti-H3K27me3 (Diagenode, cat# C15410069, lot# A1821D, polyclonal, dilution 1/500), and anti-H3K27ac (Abcam cat# ab4729, lot# GR150-367-2, polyclonal, dilution 1/500), anti-H3K36me2 (Abcam cat# ab1220 lot# GR75522, monoclonal, dilution 1/500). After incubation, membrane was washed 3 times for 10 minutes in TBST. It was incubated with secondary antibody (peroxidase conjugated, goat purified anti-rabbit IgG [Pierce cat# 31460, lot# HB987318, polyclonal]) diluted 1/5000 in TBST for 1 hour. After washing 3 times for 10 minutes in TBST, the detection was carried out using the ECL reagents and the ChemiDoc MP Imaging system (BioRad). Estimation of the relation between number of amino acid residues and molecular weight in kD was done with Calctool.

### Coral nuclei isolation

Our chromatin extraction protocol is based on Cosseau & Grunau (2011), but had to be adapted so that salinity, ionic strength and osmolarity of various buffers would match those of seawater (see Table 2 for the exact composition of all buffers). *P. acuta* (healthy or bleached), *M. digitata*, *S. pistillata* and *E. divisa* polyps were removed from the aragonite skeleton using an Airpick. This instrument is custom-designed to gently blow air through a pipet tip. Advantage of the Airpick is that it can be used with small volume of extraction solution and with a low amount of biological material. Extractions with Airpick were performed on ice for 5 minutes (or until no tissues were left on the skeleton) with the coral fragments (approximately 5 mm of diameter and 0.5 g each) inside a 50 mL tubes or small sealed plastic bags filled with 10 mL of a buffer 1 (see Table 2 for composition of all the buffers used in this article). Tubes were then centrifuged at 800 g at 4°C for 10 minutes. Supernatant was carefully removed, and pellets were gently resuspended in 1 mL of buffer 1 and 1 mL of buffer 2. The mix was transferred in Dounce homogenizer and ground on ice for 4 minutes with pestle A, and then left to rest on ice for 7 minutes. Liquid was transferred on corex tubes already containing 8 mL of buffer 3, in a manner that the homogenate would form a layer on top of buffer 3. The two solutions have a different density, which allow them to stay one on top of the other without mixing. Corex tubes were centrifuged 20 mins at 4°C and 7,800 g, with the lowest break speed possible. This step allows separating *P. acuta* nuclei, which will form a pellet at the bottom of the tube, from cell debris and Symbiodiniaceae cells, which stay at the interphase between the two buffers. Supernatant was completely removed by pouring out of the tubes and then by aspiration with micropipette. Pellets, containing coral nuclei and chromatin, were used for chromatin digestion (with the objective of using digested chromatin for ChIP).

200 μg of *Aiptasia sp*. and *A. manjano* samples were directly ground in Dounce homogenizer with 1 mL of buffers 1 & 2, and then processed the same way as *P. acuta*.

**Table 2.** Buffer composition. Final concentration for all components of the various buffers used in the chromatin extraction and micrococcal nuclease (MNase) digestion.

	Buffer 1	Buffer 2	Buffer 3	MNase Buffer
KCl	30 mM	30 mM	30 mM	
NaCl	500 mM	500 mM	500 mM	500 mM
MgCl _2_	2.5 mM	2.5 mM	2.5 mM	40 mM
Tris/Cl pH 7.4	15 mM	15 mM	15 mM	10 mM
Sucrose	1 M	1 M	1.5 M	1 M
Sodium buryrate	5 mM	5 mM	5 mM	5 mM
Dithiotreitol (DTT)	25 mM	25 mM	25 mM	
Phenylmethanesulfonyl fluoride (PMSF)	0.5 mM	0.5 mM	0.5 mM	0.1 mM
cOmplete protease inhibitor (Roche cat #11836145001)	1 X	1 X	1 X	
NP-40		0.3 %		
CaCl _2_				10 mM

### Chromatin digestion

Nuclei pellets (bleached and healthy *P. acuta*, *A. pallida* and *A. manjano*) from the coral nuclei isolation step were resuspended in 1.5 mL of MNase digestion buffer (see Table 2 for composition).

Twenty microliters of the solution were kept for a coral nuclei and Symbiodiniaceae count (see step below). Aliquots of 250 μL were prepared in Eppendorf tubes for digestion. One μL of 1 U/μL MNase (Affymetrix cat# 70196Y) was added to each tube, and incubation at 37°C was done for 0, 2, 4, 6 and 8 minutes. We also prepared a negative control with no MNase and incubated it at 37°C for 8 minutes. Because *Aiptasia* sp., *A. manjano*, *M. digitata*, *E. divisa* and *S. pistillata* were used with a comparison purpose, we only performed the digestion with three time points: 0, 4 and 8 minutes. Digestion was stopped with the addition of 20 μL of 1M EDTA and tubes were left on ice for 5 minutes. The soluble fraction of chromatin was separated from cell debris by centrifugation at maximum speed (13,000 g) 10 minutes at 4°C, and DNA in the supernatant was purified using QIAquick PCR Purification kit (Qiagen cat# 28104) following the manufacturer’s instructions. DNA fragments were eluted in 30 μL of EB buffer (a Tris-EDTA buffer supplied with the kit). Twenty to thirty microliters of the purified DNA fragments were then separated by electrophoresis through a 1.8% agarose gel stained with ethidium bromide for 60 minutes at 100 V (in 0.5X Tris Borate EDTA buffer).

### Estimation of *P. acuta* nuclei and intact Symbiodiniaceae in chromatin pellets

The chromatin extraction method we used was gentle enough to break *P. acuta* cytoplasmic membrane without lysing their nuclei or Symbiodiniaceae cells. Coral nuclei were then isolated from cell debris and Symbiodiniceae due to the density difference of the solutions used during the centrifugation step in Corex tubes (step above). To get an estimation of the amount of possible Symbiodiniaceae contamination in the nuclei/chromatin pellet when using healthy *P. acuta* as starting material, we took 20 μL of coral nuclei resuspendended in MNase digestion buffer and added Hoechst 33342 (Invitrogen cat# C10329) at a final concentration of 1/2,000. We put 10 μL on a Thoma cell counting chamber and observed on a fluorescent microscope (Leica DMLB) with objective PL Fluotar 40x and 100x at 350 nm. Hoechst 33342 stains coral nuclei with a blue fluorescence, but does not enter intact Symbiodiniaceae, which display a red fluorescence at 350 nm due to their chlorophyll content.

## Results

### Coral nuclei are isolated with less than 2% contamination of Symbiodiniaceae

Observation under fluorescent microscope showed that coral nuclei were not degraded and that there was little cell debris. We counted the number of intact Symbiodiniaceae (which have a red fluorescence at 350 nm because of their chlorophyll content) and intact coral nuclei (with blue fluorescence caused by Hoechst 33342 binding to DNA). We observed the result of 3 different nuclei isolation and never found more than 2 intact Symbiodiniaceae for every 98 coral nuclei (2%) (underlying data, supplementary file 3, Roquis *et al.*, 2021). The same result was observed in trials with *Aiptasia sp.* We observed, again through microscopy, that intact Symbiodiniaceae are stucked at the interphase between buffer 2 and buffer 3 during the centri-filtration, and do not pellet at the bottom of the tube with intact coral nuclei/chromatin. As expected, we did not find Symbiodiniaceae in menthol-bleached coral samples. The low amount of contamination with Symbiodiniaceae was confirmed by whole genome bisulfite sequencing (WGBS), in another experiment from our group using the extraction technique presented in this article: less than 0.004% of the total reads aligned to the *S. minutum* and *S. kawagutii* genomes, which are known to compose the Symbiodiniaceae clades in our *P. acuta* population ( Vidal-Dupiol *et al.*, 2019).

### *P. acuta* possesses histones, but displays an unusual chromatin structure that is also found in *E. divisa* and *A. manjano*

Protein sequences for the four core histones were found in *P. acuta* genome ( Vidal-Dupiol *et al.*, 2019) and were aligned to other species to see similarity in amino acid sequences (underlying data, supplementary file 1, Roquis *et al.*, 2021). *P. acuta* H3 and H4 have 100% and 99% identity in their amino acid sequences with *M. musculus*. Other two core histones H2A and H2B have respectively 87% and 81% of identity with *M. musculus*. Histone tails for H3 was completely identical between *M. musculus* and *P. acuta*, while a single difference (valine at position 21 was substituted by an isoleucine) was observed in the histone tail of H4. This modification of histone H4 tail was also present in *P. damicornis*, *A. digitifera*, *H. vulgaris* and *N. vectensis*. Sequence similarities were high for the four histones when compared those of the blood fluke *S. mansoni*, and similarities increased when sequence were compared with more phylogenetically related organisms ( *H. vulgaris* and *N. vectensis*). Histones H2A, H3 and H4 are completely identical between *P. acuta* and *P. damicornis* (a very similar species of the same genus), while the histone H2B amino acid sequence has 98% of identity between the two species. When compared to those of *A. digitifera*, another hermatypic scleractinian coral, 98% identity was found for *P. acuta* H2A protein, 87% with H2B, 97% with H3 and 100% with H4 (underlying data, supplementary file 1, Roquis *et al.*, 2021).

**Figure 1.**
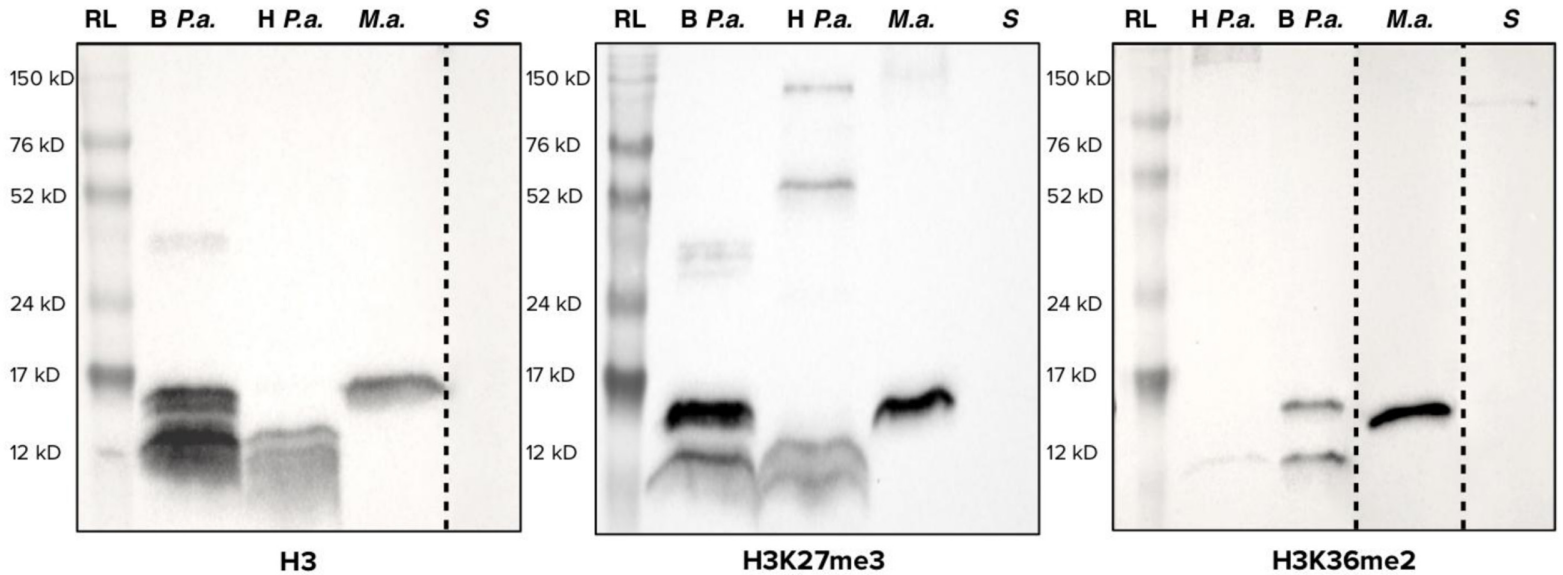
Western blots using antibodies targeting histone H3 (left), trimethylation of lysine 27 of histone H3 (H3K27me3, center) and dimethylation of lysine 36 of histone H3 (H3K36me2, right). RL = Amersham ECL™ Full-Range Rainbow Marker; B *P. a*. = Bleached *Pocillopora acuta*; H *P. a*. = Healthy *Pocillopora acuta*; *S* = Symbiodiniaceae; *M.a.* = *Mesocricetus auratus*. For maximum readability, some lanes were cropped (shown with dashed lines), brightness and contrast were increased for H3K36me2. Original, uncropped and unedited, pictures of these western blots are available in the underlying data, supplementary file 2 ( Roquis *et al.*, 2021).

We then performed western blots to determine if commercial antibodies targeting histone modifications could be used on *P. acuta*. We tested hamster brain (positive control), crude protein extract of free Symbiodiniaceae, and bleached corals without Symbiodiniaceae. Symbiodiniaceae extract was used as a negative control, as their core histone sequences, and particularly their lateral chains, are fairly different from metazoan ( Marinov & Lynch, 2015), and previous attempt by our group to detect them with commercial antibodies targeting histone modifications were always unsuccessful. Results of the western blots are shown on Figure 1. 

Results for the three antibodies (anti-H3, anti-H3K27me3, anti-H3K36me2) were consistent between organisms. In all three cases, no signal was detected for Symbiodiniaceae *,* and a signal at the expected size (approximately 15 kDa) for histone H3 was observed in hamster. In healthy corals, a signal was observed, but at 12 kDa. Bleached coral displayed both the 12 kDa band and the expected 15 kDa band. The difference of about 3 kD corresponds roughly to a difference of 25 amino acid residues in size, reminiscent of histone H3 clipping ( Azad *et al.*, 2018; Santos-Rosa *et al.*, 2009).

**Figure 2.**
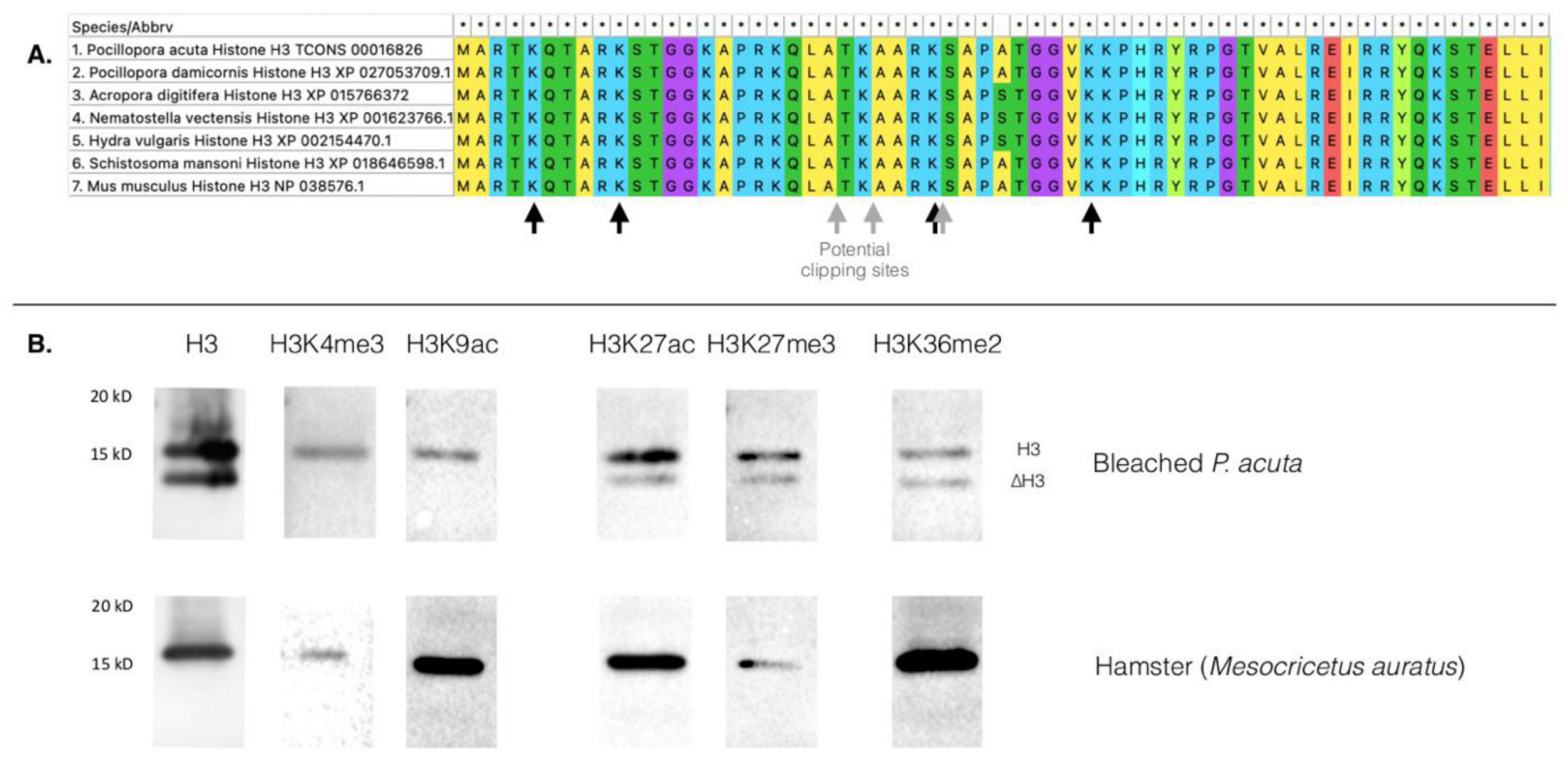
**A**. Partial amino acids alignment of histone H3 (see underlying data, supplementary file 1 for the complete alignment, Roquis *et al.*, 2021). Black arrows point at amino acid positions where chemical modifications are targeted by the antibodies we used (lysines at positions 4, 9, 27 and 36). Grey arrows potential histone H3 clipping sites based on literature data for other organisms. An asterisk (*) means that the amino acid is conserved between all species **B**. Western blots using antibodies targeting histone H3, trimethylation of H3 lysine 4 and 27 of histone H3 (H3K4me3, H3K27me3), acetylation of H3 lysine 9 and lysine 27 (H3K9ac, H3K27ac), and dimethylation of lysine 36 of histone H3 (H3K36me2). Hamster *Mesocricetus auratus* is used as a positive control. For maximum readability, some lanes were cropped. Original, uncropped, pictures of these western blots are available in the underlying data, supplementary file 2 ( Roquis *et al.*, 2021).

To narrow down the clipping site, we used three more antibodies (anti-H3K4me3, anti-H3K9ac and anti-H3K27ac) on acid-extracted purified histones and found the clipping to occur between amino-acid position 9 and 27 ( Figure 2).

**Figure 3.**
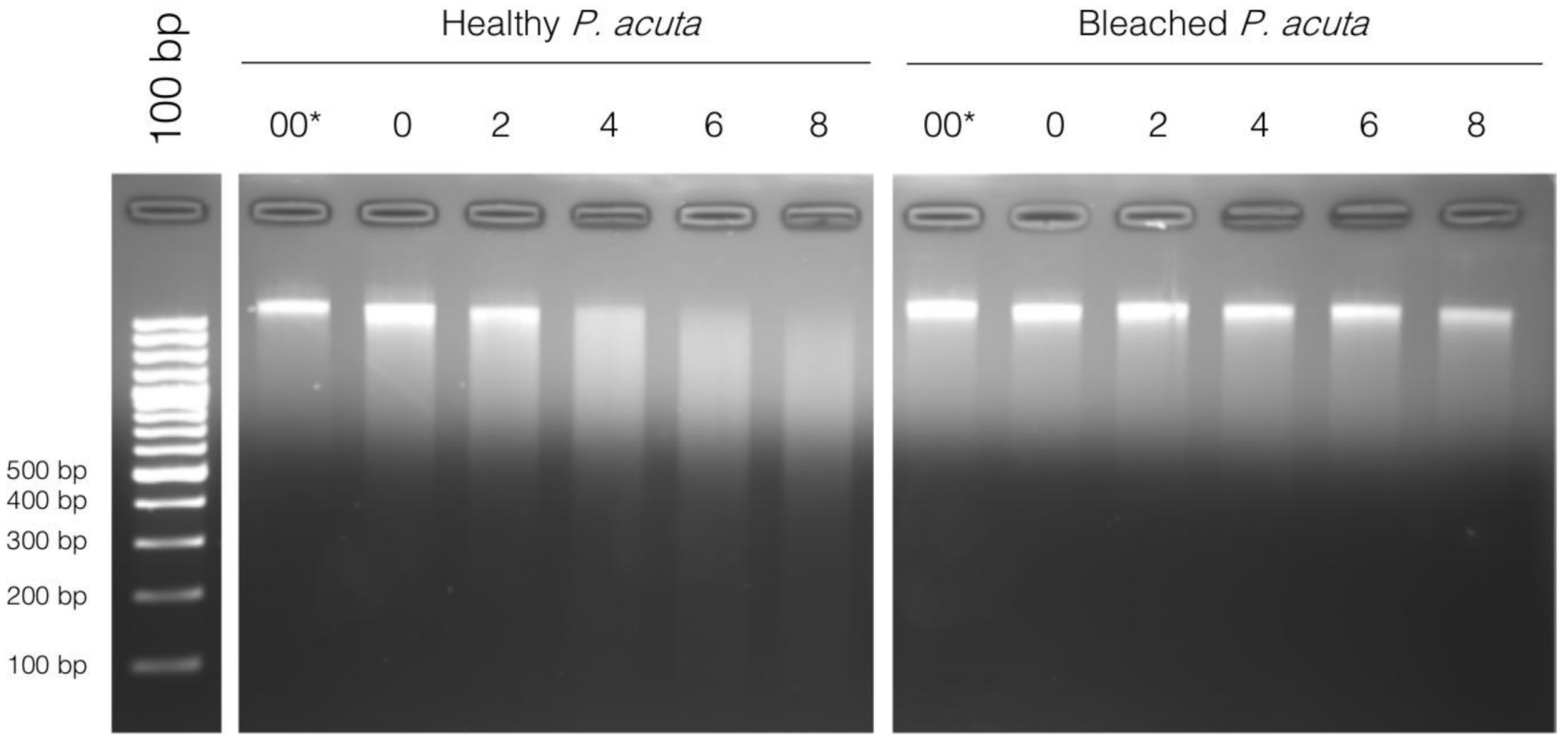
*Micrococcal* nuclease (MNase) digestion profile of chromatin from healthy and bleached *Pocillopora acuta* in function of digestion time (in minutes) at 37°C, ranging from 0 minutes to 8 minutes. 00 corresponds to chromatin left at 37°C for 8 minutes without the addition of MNase, and 0 is addition of MNase immediately followed by incubation on ice with EDTA (enzyme inhibitor). 100 bp DNA ladder (Promega cat# G2120) is used for band size estimation. Uncropped gel pictures are available in the underlying data, supplementary file 2 ( Roquis *et al.*, 2021).

From these results, we suspected that there could be a different chromatin structure between bleached and healthy *P. acuta*, and we carried out a chromatin digestion with micrococcal nuclease (MNase). MNase digests DNA between nucleosomes, which creates DNA fragments with sizes that are multiples of approximately 150 bp (length of DNA wrapped around a nucleosome). When separated on a gel, the digested DNA looks like a ladder. After a prolonged digestion, only 150 bp fragments, corresponding to a mononucleosomal digestion would be detectable on an agarose gel. A very good example of this can be seen in Hewish & Burgoyne (1973). We did not observe this type of result for neither healthy nor bleached *P. acuta*. Instead. DNA is completely degraded with time, and no nucleosomal size fragments are seen ( Figure 3).

**Figure 4.**
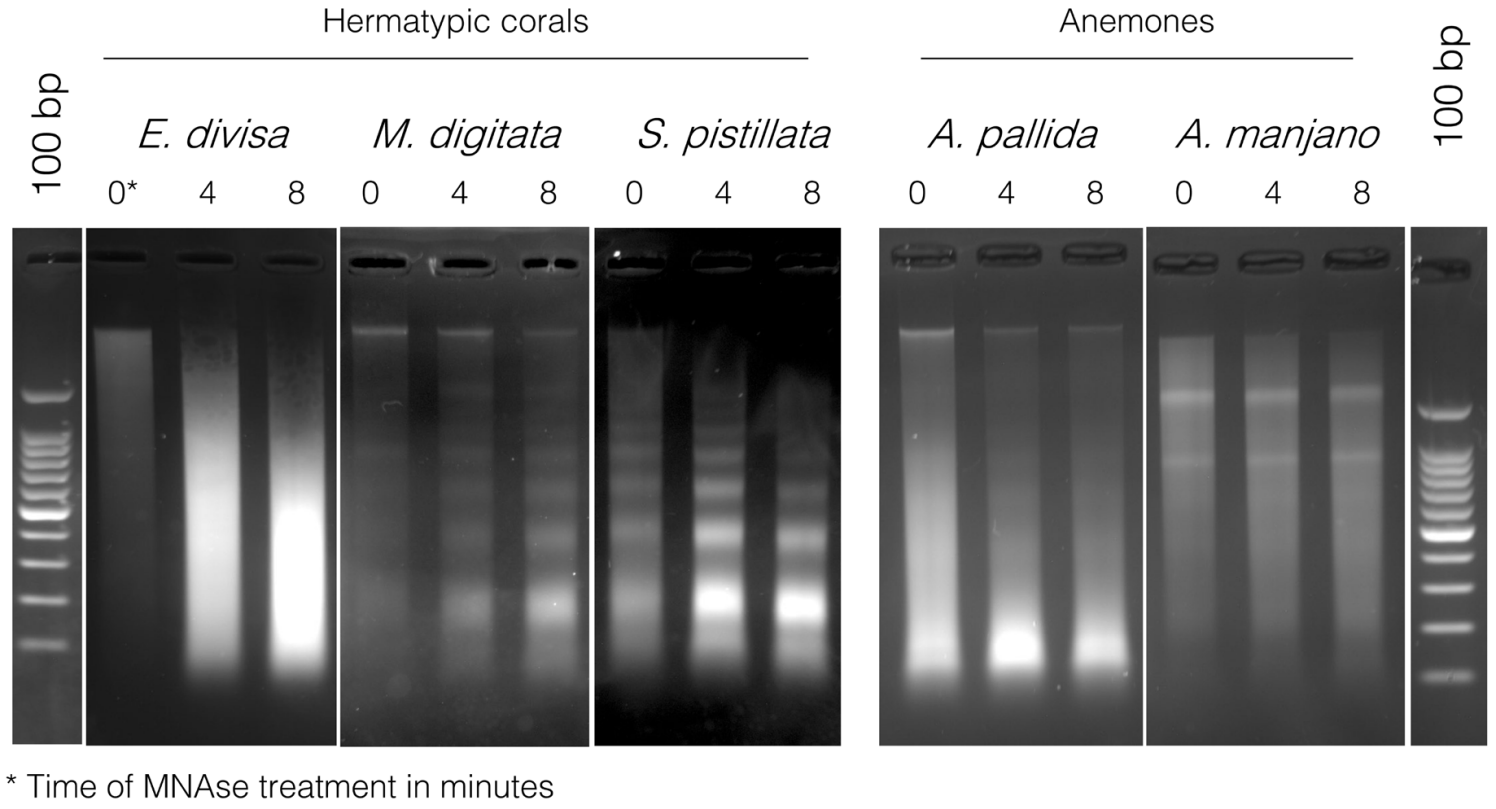
Micrococcal nuclease (MNase) digestion profiles for three hermatypic corals, E *uphyllia divisa*, *Montipora digitata* and *Stylophora pistillata*, as well as for the two sea anemones *Aiptasia sp*. and *Anemonia manjano*. 100 bp DNA ladder (Promega cat# G2120) is used for band size estimation. Uncropped gel pictures are available in the underlying data, supplementary file 2 ( Roquis *et al.*, 2021).

To verify that it was not a problem with the chromatin extraction and digestion technique, we performed the same experiment on three other hermatypic corals *E. divisa*, *M. digitata* and *S*. *pistillata*, as well as two symbiotic anemones, *Aiptasia sp.* and *A. manjano.* As it can be seen on Figure 4, *E. divisa* and *A. manjano* present the same unusual digestion profile as *P. acuta*, while *S. pistillata*, *M. digitata* and *Aiptasia sp.* show the expected digestion profile, in the shape of a ladder and with an enrichment at 150 bp (corresponding to mononucleosomal fragments). The ladder profile is not striking in *A. pallida* (probably because of an excess of starting biological material), but enrichment around 150 bp is clearly visible.

## Discussion

**Figure 5.**
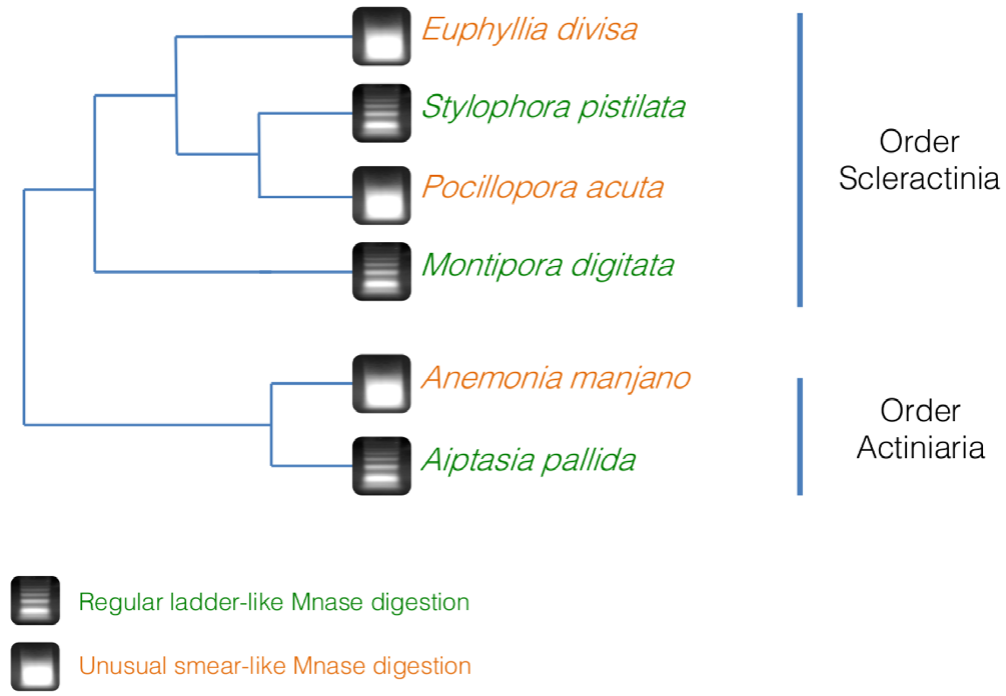
Simplified phylogeny of the coral and anemone species used in this study, based on Daly *et al.* (2003) and Romano & Palumbi (2009) and associated with the results of the micrococcal nuclease (MNase) digestion. The digestions profiles do not follow the phylogeny, hinting that the unusual chromatin structure profile is not an ancestral conserved trait in the Hexacorallia subclass.

In this article, we describe (i) a straightforward method to separate *P. acuta* chromatin and DNA from Symbiodiniaceae material, (ii) we provide the first description of *P. acuta* core histones, and (iii) we show that bleaching influences histone H3 structure in *P. acuta*. Alignments of protein sequences of histones H2A, H2B, H3 and H4, which compose nucleosomes, show that they are well conserved in *P. acuta*, with only little difference to mice. Interestingly, we did not observe the histone H3 band at the expected size (15 kDa) in healthy corals for any of the three antibodies we used (anti-H3, anti-H3K27me3, anti-H3K36me2). Instead, a single band of lower molecular weight (12 kDa ∆H3) is visible. Bleached corals are different, with both the expected H3 band at 15 kDa, and the truncated one at 12 kDa. The size difference of the bands suggests clipping of H3 tails at roughly 25 residues. Clipping after alanine at position 21 (Ala21) in histone H3 was observed in *Saccharomyces cerevisiae*. Cleavage activity is induced under conditions of nutrient deprivation and sporulation ( Santos-Rosa *et al.*, 2009). In the human parasite *Plasmodium falciparum*, H3 clipping occurs after Ala21 and is abundant in genes regulating DNA replication of the pathogen ( Herrera‐Solorio *et al.*, 2019). H3 cleavage was also observed in vertebrates where it is cell type-dependent and plays multiple important biological functions ( Azad *et al.*, 2018). Two clipping sites are documented, one between lysine at position 21 (Lys-23) and Alanine at position 24 (Ala-24) and the other between lysine at position 27 (Lys-27) and serine at position 28 (Ser-28) ( Mandal *et al.*, 2013). Since we obtained a positive signal on Western blots for ∆H3 with anti-H3K27me3, cleavage occurs downstream of position 27 in *P. acuta*. Mass spectrometry will be a next step to better identify the clipping site. Histone clipping is probably involved in the regulation of gene expression in a multitude of processes and is suspected to have a direct effect on chromatin structure ( Azad *et al.*, 2018; Zhou *et al.*, 2014). Surprisingly, ∆H3 is the major H3 isoform in healthy corals as opposed to bleached ones. We did not observe any signal in the Symbiodiniaceae extract, which confirms that the low molecular weight band (12 kDa ∆H3) we observe in healthy and bleached coral truly comes from *Pocillopora* and not from its symbiotes. It was thought for some time that Symbiodiniace did not possess real histones ( Rizzo, 2003), but recent work demonstrated that they do have core histones, but usually of larger size and serving other functions than DNA packing and chromatin structure ( Bayer *et al.*, 2012; Marinov & Lynch, 2015; Nand *et al.*, 2021; Roy & Morse, 2012). As we suspected, their protein structure is too different to be detected on Western blots by our commercial antibodies. Symbiodiniaceae live within coral cells, and their presence or absence may cause major cellular and nuclear reorganization as they induce significant transcriptome remodeling ( DeSalvo *et al.*, 2010). There is the possibility that *P. acuta* chromatin may be compacted in different fashion, with other histone forms or histone-like proteins, depending on its symbiotic state. Recently, ( Török *et al.*, 2016) have shown that *Hydractinia echinata*, another Cnidaria, possesses 14 different histones (different from the canonical H1.1, H2A.1, H2B.1, H3.1 and H4.1 histones), including a novel H3.3.2 variant. These histones variants are expressed at different life stages or in diverse tissues to efficiently pack DNA. It is plausible that a similar diversity exists in *P. acuta,* which should be investigated. Further analyses are needed to confirm these hypotheses and to investigate if this phenomenon is also present in the other corals and anemone species used in this study. Surprisingly, treatment of the chromatin of *P. acuta* (healthy or bleached), *E. divisa* and *A. manjano* with MNase did not produce nucleosomal fragments. MNase cuts preferentially between nucleosomes because of steric hindrance. DNA is wrapped around nucleosomes for a 150 bp length, and incomplete digestion present a “ladder” profile on gels, with each band being a multiple of 150 bp, while complete digestion shows a unique, intense band at 150 bp ( Hewish & Burgoyne, 1973; Hörz & Altenburger, 1981; Keene & Elgin, 1981; Noll & Kornberg, 1977; Wu *et al.*, 1979; Zaret, 2005). In these three species, DNA is completely digested by MNase ( Figure 3 & Figure 4), similarly to what would happen in absence of nucleosomes ( Hewish & Burgoyne, 1973). On the other hand, *Aiptasia sp.*, *M. digitata* and *S. pistillata*, gave the expected digestion profile for MNase. These results indicate that some species within the Hexacorallia subclass have an unusual chromatin structure in which histones might be replaced by other proteins, or where nucleosomes have a different or weaker bond to DNA than what is commonly observed in other metazoan. However, these two digestion profiles are not coherent with the phylogeny of the species we used in this study ( Daly *et al.*, 2003; Romano & Palumbi, 2009), implying that it is not a feature specific of an order or a genus ( Figure 5). At the moment, we cannot state if this unusual chromatin structure is an ancestral feature of the Hexacorallia, which was lost in some species, or if it is a new character that appeared at multiple times within the Hexacorallia subclass. More analyses are needed in order to see if this unusual chromatin structure is limited to some species in the Scleractinia and Actinaria orders, or if it is more widespread within Hexacorallia subclass or Anthozoa class.

## Conclusion

All together the results provided in this work represent a first inside into the chromatin, nucleosome and histone structure of *P. acuta.* The unusual patterns highlighted in this study and partly shared with other cnidarian will need to be further studied in order to better understand its role and function in corals. This understanding will be necessary to address the essential question about the role of epigenetic modifications in rapid adaptation of these endangered organisms ( Torda *et al.*, 2017).

## Data availability

### Underlying data

Zenodo: Suppl. Information to "The tropical coral Pocillopora acuta displays an unusual chromatin structure and shows histone H3 clipping plasticity upon bleaching". https://doi.org/10.5281/zenodo.5121858 ( Roquis *et al.*, 2021).

This project contains the following underlying data:

- Supplementary File 1.pdf (Amino acids sequence alignments for the four core histones [H2A, H2B, H3, H4] in six species: *Pocillopora acuta*, *Acropora digitifera*, *Nematostella vectensis*, *Hydra vulgaris*, *Schistosoma mansoni* and *Mus musculus*.)
- Supplementary File 2.pdf (Original [uncropped and unedited] images used for Figures 1 to 4.)
- Supplementary File 3.xlsx ( *P. acuta* nuclei and Symbiodiniaceae count on a Thoma cell counting chamber done over three different nuclei extractions.)

Data are available under the terms of the Creative Commons Attribution 4.0 International license (CC-BY 4.0).
