# Supplementary material for: The tropical coral Pocillopora acuta displays an unusual chromatin structure and shows histone H3 clipping plasticity upon bleaching
Source: Wellcome Open Res. 2022 Jan 25;6:195. Originally published 2021 Jul 29. [Version 2] doi: 10.12688/wellcomeopenres.17058.2 (PMC8889044; doi:10.12688/wellcomeopenres.17058.2)
Supplement: Peer review history

# Peer review: Reviewer response for version 2

**DOI:** 10.21956/wellcomeopenres.19483.r48241

**Published:** 2022-03-01

**Authors:** Paola Furla, Eric Gilson

**Affiliations:** 1 CNRS, INSERM, Institute for Research on Cancer and Aging (IRCAN), Côte d'Azur University, Nice, France

We acknowledge that the authors took into account our reserves in the interpretation of some the results in the discussion of their results. Therefore, we consider that the revised manuscript can be published.

Is the work clearly and accurately presented and does it cite the current literature?

Yes

If applicable, is the statistical analysis and its interpretation appropriate?

Yes

Are all the source data underlying the results available to ensure full reproducibility?

Yes

Is the study design appropriate and is the work technically sound?

Yes

Are the conclusions drawn adequately supported by the results?

Partly

Are sufficient details of methods and analysis provided to allow replication by others?

Yes

Reviewer Expertise:

Coral symbiosis, biochemical signaling, genome stability

We confirm that we have read this submission and believe that we have an appropriate level of expertise to confirm that it is of an acceptable scientific standard.

# Peer review: Reviewer response for version 1

**DOI:** 10.21956/wellcomeopenres.18839.r46367

**Published:** 2021-11-22

**Authors:** Paola Furla, Eric Gilson

**Affiliations:** 1 CNRS, INSERM, Institute for Research on Cancer and Aging (IRCAN), Côte d'Azur University, Nice, France

This study focuses on the chromatin structure of the coral *P. acuta*. Overall, this study paves the way, both methodologically and conceptually, for future studies aimed at understanding coral chromatin plasticity facing environmental changes, a clear frontier in our knowledge of coral physiology.

 As inferred from genomic data, the conservation of the four histones is very high in *P. acuta *(and other coral species), and almost identical to those of other animals, which is a known property of histone gene evolution.

 The authors successfully used a method of host nucleus isolation (with less than 2% contamination with the symbionts).

 By Western blotting, they show that the use of commercial antibodies, in particular those raised against post-translational modifications, are useable in coral studies. Interestingly, they observed that the full length H3 is seen only in bleached extract suggesting an H3 clipping processing in healthy colonies that is impaired upon bleaching.

 By micrococcal digestion, no classical ladder of nucleosomes is visualized, suggesting a particular chromatin organization in this coral species.

 Overall, the results are interesting, but the authors should be more prudent in their conclusions.

**Specific comments:**

- The authors could mention whether they detect histone gene clusters and histone variants in the coral genome and to what extent this echoes the situation in humans and mice.
- Figure 1: The authors should be more prudent regarding in the interpretation regarding clipping since the result could be explained by a differential protease activity during the extraction of bleached and healthy colonies
- Since the authors identified antibodies that differentially recognized the clipped and full length forms of H3, the authors could perform immunofluorescence experiments with these diagnostic antibodies on bleached and healthy coral nuclei to confirm that indeed the majority of H3 is clipped in healthy nuclei.
- The authors should more prudent regarding the interpretation of the negative results with micrococcal digestion. The fact that they were successful to produce a nucleosome ladder with other corals (e.g. *S. pistillata*) does not mean that, with their *P. acuta *nuclei, there are no extraction or digestion problems that prevent proper micrococcal nuclease digestion. A possible way to test that would be to mix *P. acuta* and *S. pistillata *nuclei prior to nuclease digestion and to analyze the ladder by differential hybridization on Southern blotting (assuming that differential probes exist between the two coral species). In any case, the authors should be more balanced in their conclusion.

Is the work clearly and accurately presented and does it cite the current literature?

Yes

If applicable, is the statistical analysis and its interpretation appropriate?

Yes

Are all the source data underlying the results available to ensure full reproducibility?

Yes

Is the study design appropriate and is the work technically sound?

Yes

Are the conclusions drawn adequately supported by the results?

Partly

Are sufficient details of methods and analysis provided to allow replication by others?

Yes

Reviewer Expertise:

Coral symbiosis, biochemical signaling, genome stability

We confirm that we have read this submission and believe that we have an appropriate level of expertise to confirm that it is of an acceptable scientific standard, however we have significant reservations, as outlined above.

# Response

**Published:** 2022-01-15

**Authors:** Christoph Grunau

**Affiliations:** University of Perpignan Via Domitia, France

**We are grateful towards the reviewer for taking the time to read our manuscript and providing the very constructive comments. We found that the comments were really pertinent and offer possibility of experiments we shall do in the future. **

- The authors could mention whether they detect histone gene clusters and histone variants in the coral genome and to what extent this echoes the situation in humans and mice.

**Initially, this was our goal at the beginning of our bioinformatics analyses on *P. acuta* genome. Unfortunately, the quality of the draft genome we have for *P. acuta* is low, and we observed that many loci with repetitive regions, including hypothetical histone variants, are often misassembled or truncated. We deemed not feasible with the current genome quality to properly list and compare known histone variants orthologs in *P. acuta*, and we chose to focus only on the canonical core nucleosomal histones as we had sufficient RNA-seq evidences to confirm we had the proper sequences for these four proteins. The *P. acuta* genome is still a work in progress, and we will definitely revisit this analysis once the overall quality will be improved. **

- Figure 1: The authors should be more prudent regarding in the interpretation regarding clipping since the result could be explained by a differential protease activity during the extraction of bleached and healthy colonies

**We believe that the presence of protease inhibitors in the extraction buffers impede any protease activity. Both bleached and healthy corals were extracted several times, and the clipping phenomenon was constantly reproducible only in health corals. We have included this into the manuscript.**

- Since the authors identified antibodies that differentially recognized the clipped and full-length forms of H3, the authors could perform immunofluorescence experiments with these diagnostic antibodies on bleached and healthy coral nuclei to confirm that indeed the majority of H3 is clipped in healthy nuclei.

**The reviewer is right that many further experiments will be necessary to investigate in more detail the location and the function of histone clipping in corals. The experiments that were suggested are entirely justified and will answer some of the questions that remain to be answered in the future. Nevertheless, we believe to have clearly established that there is indeed differential clipping and we hope to have convinced the reviewer about at least this. We have added a comment about these open questions in the manuscript. **

- The authors should more prudent regarding the interpretation of the negative results with micrococcal digestion. The fact that they were successful to produce a nucleosome ladder with other corals (e.g. *S. pistillata*) does not mean that, with their *P. acuta *nuclei, there are no extraction or digestion problems that prevent proper micrococcal nuclease digestion. A possible way to test that would be to mix *P. acuta* and *S. pistillata *nuclei prior to nuclease digestion and to analyze the ladder by differential hybridization on Southern blotting (assuming that differential probes exist between the two coral species). In any case, the authors should be more balanced in their conclusion.

**We agree with the reviewer on this aspect. Our initial hypothesis when we observed the smeared MNase digestion pattern in *P. acuta* was that something went wrong with the chromatin extraction. We did not mention it in the manuscript, but we tried 24 different types of chromatin extractions on *P. acuta*, and always obtained the same digestion profile with the MNase (documented in** **https://tel.archives-ouvertes.fr/tel-01265805** **at page 273), which then convinced us that *P. acuta’s* chromatin was very likely to have a different structure. We included this information in the manuscript.**

# Peer review: Reviewer response for version 1

**DOI:** 10.21956/wellcomeopenres.18839.r45225

**Published:** 2021-08-20

**Authors:** Maarten Dhaenens

**Affiliations:** 1 Laboratory for Pharmaceutical Biotechnology, University of Ghent, Ghent, Belgium

In “The tropical coral Pocillopora acuta displays an unusual chromatin structure and shows histone H3 clipping plasticity upon bleaching”, Roquis *et al. *describe their pioneering efforts in studying histones and to a wider extend the chromatin structure in (bleaching) coral. While still somewhat rudimentary in experimental design and techniques applied, the manuscript straightforwardly describes a novel and rather exciting finsing on H3 clipping in coral.

 Because of the limited amount of experiments presented, there is little to object to the way they were presented and interpreted. Indeed, a third wave of histone clipping manuscripts is currently being published (Dhaenens, M., EMBO Reports, 2021) ^1^ and especially histone H3 clipping is no longer a curiosity in the side line. While I do not want to promote self-citation, I do believe that our 2015 review on histone clipping and degradation really describes the importance of understanding this mechanism in as many organisms as possible. ^2^

 I have no specific comments on this publication, apart from being eager to seeing this unfold with more MS-based and possibly microcopy-based follow up experiments. If (the absence of) histone clipping turns out to play an import role in coral bleaching, trying to stimulate the protease activity (which is still not revealed for coral and could turn out be yet another addition to the already long list of proteases) could even turn out be beneficial and thus important for the future of marine sea life.

Is the work clearly and accurately presented and does it cite the current literature?

Partly

If applicable, is the statistical analysis and its interpretation appropriate?

Not applicable

Are all the source data underlying the results available to ensure full reproducibility?

Yes

Is the study design appropriate and is the work technically sound?

Yes

Are the conclusions drawn adequately supported by the results?

Yes

Are sufficient details of methods and analysis provided to allow replication by others?

Yes

Reviewer Expertise:

Histone research; histone clipping; mass spectrometry; data-independent acquisition technology

I confirm that I have read this submission and believe that I have an appropriate level of expertise to confirm that it is of an acceptable scientific standard.

## References

[1] : Histone clipping: the punctuation in the histone code. *EMBO Rep* .2021;22(8) : 10.15252/embr.202153440 e53440 10.15252/embr.202153440 34232560PMC8344887

[2] : Histone proteolysis: a proposal for categorization into 'clipping' and 'degradation'. *Bioessays* .2015;37(1) : 10.1002/bies.201400118 70-9 10.1002/bies.201400118 25350939PMC4305269
